# The BioJS article collection of open source components for biological data visualisation

**DOI:** 10.12688/f1000research.3-56.v1

**Published:** 2014-02-13

**Authors:** Manuel Corpas

**Affiliations:** 1 The Genome Analysis Centre, Norwich Research Park, Norwich, NR4 7UH, UK

## Abstract

Data-driven research has gained momentum in the life sciences. Visualisation of these data is essential for quick generation of hypotheses and their translation into useful knowledge. BioJS is a new proposed standard for JavaScript-based components to visualise biological data. BioJS is an open source community project that to date provides 39 different components contributed by a global community. Here, we present the BioJS *F1000Research* collection series. A total of 12 components and a project status article are published in bulk. This collection does not intend to be an all-encompassing, comprehensive source of BioJS articles, but an initial set; future submissions from BioJS contributors are welcome.

## Commentary

Journal articles are a useful source for dissemination of knowledge and attribution of recognition. The *F1000Research* BioJS article collection provides a sample of BioJS components contributed by the community to date. Articles in this series follow a similar structure to the ones published by the *Bioinformatics* Journal *Application Notes*. A description of the component’s functionality, design attributes and potential applications are included in each component article of this collection. Each BioJS component article is about two pages long and follows a structure describing its design and implementation, a use case, and potential applications for the component. The use case includes, at least, a figure with a biologically meaningful example and original biological insight. We do not expect every BioJS component to be published in this series; we do encourage, however, all interested BioJS developers to submit articles for peer review if they so wish.

In this initial set of publications we present a) a community article in which we describe the status of the project in 2014 ^1^ and b) a select group of components available through the BioJS registry ( http://www.ebi.ac.uk/Tools/biojs/registry/). Components published in this series can be tested in the BioJS registry, with no need to install them first. Components presented in this collection can be grouped according to the type of data they are designed to visualise. According to this criterion, there are components used to visualise sequences, proteins, DNA, networks and phylogenetic trees. There are also some components that visualise a specific type of data linked to a resource. For instance, some widgets use specific data from InterMine ^2^, PSICQUIC ^3^ or KEGG ^4^. Among those components purposely built to render data visualisations for a particular a resource, we present *KEGGViewer* ^5^, a component that visualises KEGG pathways; *PsicquicGraph* ^6^, which visualises molecular interactions from PSICQUIC servers, and the *InterMine List Analysis* ^7^ and *Table* ^8^ components, which display statistical analyses and a dynamic result table, respectively, for InterMine-compatible data. Among components not designed for a particular resource, the collection includes *FeatureViewer* ^9^, a component that lays out, maps and renders position-based annotations for protein sequences; *HeatMapViewer* ^10^, which renders matrix-formatted data; *treeWidget* ^11^, a component that visualises phylogenetic trees; a set of components to visualise protein-protein interaction networks ^12^; *DAGViewer* ^13^, a directed acyclic graph viewer with facilities for rendering ontologies; *Sequence* ^14^, a component for visualising sequences; *wigExplorer* ^15^, which visualises wig format data; and *DNAContentViewer* ^16^, which displays GC/AT content of a DNA sequence. The types of visualisation in this initial set of articles thus include network and directed acyclic graphs, a list, a table, generic features, a heat map, a phylogenetic tree and sequence-based objects.

## Summary

We present the BioJS series articles for the *F1000Research* BioJS collection. Each component presented in this collection has been previously validated and is readily available for use and testing through the BioJS registry. Every component must follow the minimum standards for documentation, architecture and deposition stated by the project ( https://docs.google.com/document/d/1gG036Bvwl4i-KX5BTHddGzeE_5eospL-864BrnsAS_s/edit). We encourage potential contributors for BioJS to submit software articles to this collection describing the functionality of valid components.
